# Pooled whole‐genome sequencing of interspecific chestnut (*Castanea*) hybrids reveals loci associated with differences in caching behavior of fox squirrels (*Sciurus niger* L.)

**DOI:** 10.1002/ece3.4336

**Published:** 2018-10-23

**Authors:** Nicholas R. LaBonte, Keith E. Woeste

**Affiliations:** ^1^ Department of Crop Sciences University of Illinois Urbana Illinois; ^2^ USDA Forest Service Northern Research Station Hardwood Tree Improvement and Regeneration Center West Lafayette Indiana

**Keywords:** coevolution, Fagales, forest regeneration, mutualism, seed dispersal

## Abstract

Dispersal of seeds by scatter‐hoarding rodents is common among tropical and temperate tree species, including chestnuts in the genus *Castanea*. Backcrossed (BC) interspecific hybrid chestnuts exhibit wide variation in seed traits: as the parent species (*Castanea dentata* and *C. mollissima*) have distinct seed phenotypes and tend to be handled differently by seed dispersers, phenotypic variation in BC trees is likely due to inheritance of genes that have undergone divergent evolution in the parent species. To identify candidate genomic regions for interspecific differences in seed dispersal, we used tagged seeds to measure average dispersal distance for seeds of third‐generation BC chestnuts and sequenced pooled whole genomes of mother trees with contrasting seed dispersal: high caching rate/long distance; low caching rate/short distance; no caching. Candidate regions affecting seed dispersal were identified as loci with more *C. mollissima* alleles in the high caching rate/ long‐distance pool than expected by chance and observed in the other two pools. Functional annotations of candidate regions included predicted lipid metabolism, dormancy regulation, seed development, and carbohydrate metabolism genes. The results support the hypothesis that perception of seed dormancy is a predominant factor in squirrel caching decisions, and also indicate profitable directions for future work on the evolutionary genomics of trees and coevolved seed dispersers.

## 1 INTRODUCTION

Successful dispersal of seeds is pivotal for a tree's evolutionary fitness because effective dispersal allows parent trees to propagate within an immediate area, may decrease density‐dependent mortality near parent trees, enables a tree's offspring to colonize distant suitable habitats (Howe & Smallwood, 1982; Janzen, 1971), expanding the species’ range, and reduces extinction risk (Larson‐Johnson, 2015; Schupp, Jordano, & Gomez, 2010; Vander Wall, 2001). Among angiosperm trees, diverse strategies for seed dispersal have evolved (Friis, Crane, & Pederson, 2011), including wind dispersal (e.g., *Acer*,* Platanus*,* Populus*), dispersal by frugivores (e.g., *Prunus*,* Diospyros*), and dispersal by granivores that cache seeds in the ground. Much of the forest canopy in eastern and central North America is, or was historically, dominated by granivore‐dispersed genera (e.g., *Quercus*,* Juglans*,* Castanea*) in the order Fagales (Leopold, McComb, & Muller, 1998). Across many tree taxa, including Fagales (Larson‐Johnson, 2015), dispersal by seed‐caching animals is actually associated with greater average dispersal distance than wind dispersal (Thomson, Moles, Auld, & Kingsford, 2011).

The coevolved relationship between trees and scatter‐hoarding seed dispersal agents is a “conditional mutualism” (Theimer, 2005): rodents and birds consume large numbers of seeds, but cache enough in the soil—sometimes at considerable distances from the mother tree—to allow a few to escape predation and germinate. This coevolution has resulted in a wide variety of seed‐packaging strategies employed by granivore‐dispersed trees—from large, hard‐shelled seeds only edible to rodents with specialized dentition (Stapanian & Smith, 1978; Tamura & Hayashi, 2008) to small nuts that can be carried and eaten by birds (Johnson & Webb, 1989; Richardson, Licthi, & Swihart, 2013). The vast majority of seeds cached by scatter‐hoarding rodents are consumed at some point (Brodin, 2010; Calhane, 1942; Tamura, Hashimoto, & Hayashi, 1999; Thompson & Thompson, 1980), so there must be a fitness advantage for cached versus noncached seeds in order for trees and squirrels to mutually benefit from scatter‐hoarding (Zwolak & Crone, 2012). Caching in the soil hides the seed from other potential predators at the surface and can reduce desiccation (Vander Wall, Kuhn, & Beck, 2005; Schupp et al., 2010; Zwolak and Crone 2012). In relatively thin‐shelled nuts such as oak and chestnut, death through desiccation is a danger for exposed seeds (Connor, Donahoo, & Schafer, 2006), so these species are likely to derive a net benefit from their relationship with scatter‐hoarding rodents.

American chestnut (*Castanea dentata*, abbreviated *Cd*) was formerly a keystone species in the forests of the Appalachian Mountains of the eastern United States and adjacent regions before its elimination by chestnut blight (*Cryphonectria parasitica*). It is currently the subject of a breeding program (led by the American Chestnut Foundation, abbreviated TACF) using several generations of backcrossing to introgress blight‐resistance genes from Chinese chestnut into the American chestnut genome. Dispersal by squirrels will be important for chestnut restoration, which is the goal of the TACF breeding program. Chestnut is likely to be planted largely in reclaimed minelands, old fields, and other open sites (Jacobs, 2007). The success of restoration based on hybrids will depend on whether hybrids are dispersed and regenerate naturally (Jacobs, Dalgleish, & Nelson, 2012). Scatter‐hoarding in the eastern North American deciduous forest is primarily practiced by two species of tree squirrels, the eastern gray squirrel (*Sciurus carolinensis*) and the fox squirrel (*Sciurus niger*), as well as the blue jay (*Cyanocitta cristata*) (Blythe, Lichti, Smyser, & Swihart, 2015; Moore, McEuen, Swihart, Contreras, & Steele, 2007; Richardson et al., 2013; Smith and Stapanian 2002; Steele, Smallwood, Spunar, & Nelsen, 2001). Although third‐generation backcross (BC3) chestnuts used for species restoration will inherit only a small fraction of the *Cm* genome, this genetic material may influence ecologically important traits (Worthen, Woeste, & Michler, 2010); for example, BC3 seeds were dispersed farther on average than seeds of *Cd* (Blythe et al., 2015). Hybrid background may affect this crucial, coevolved ecological relationship in other ways as well.

The large number of backcrossed hybrid chestnuts generated by the blight‐resistance breeding program of TACF and the publication of a Chinese chestnut draft genome (Carlson et al., 2014; Pereira‐Lorenzo et al., 2016) make backcrossed trees an interesting model for studying the genomic basis of interspecific differences in seed dispersal. Tree squirrels, including *Callosciurus erythraeus* and *Sciurotamias davidianus*, are important dispersal agents of *Cm* in its native range (Xiao, Gao, & Zhang, 2013), but *Cm* has evolved alongside a number of nut‐bearing trees (e.g., *Lithocarpus* spp, *Camelia oleifera*) that are not present in eastern North America and may have indirectly exerted selective pressure on *Cm* by competing for the attention of seed dispersers. *Cm* seeds are larger, on average, than American chestnuts, and squirrels are more likely to cache a large seed—and carry it farther—than a small one (Jansen et al., 2002; Xiao, Zhang, & Wang, 2005). In addition to nut size, squirrels are aware of physiological cues in seeds that signal the start of germination and take measures to maximize the nutritional utility of nondormant seeds (Fox, 1982; Smallwood, Steele, & Faeth, 2001; Steele et al., 2001). Dormant seeds are more likely to be cached and carried a longer distance than nondormant seeds, which are usually eaten without caching. All chestnuts go through true dormancy with a chilling period (Baskin & Baskin, 1998), so any difference in dormancy‐breaking among *Cd*,* Cm*, and hybrids would presumably be marginal. Physiological differences in the seeds of different chestnut species, however, could be interpreted by squirrels as signals pertaining to dormancy and thus influence caching (Steele et al., 2001; Sundaram et al. 2015, Sundaram, 2016).

Because phenotypic variance in seed traits can be caused by variable inheritance of alleles from *Cm* in the *Cd* genomic background of BC3s, we sought to identify loci that influence seed traits and dispersal by associating the presence or absence of *Cm* alleles in a mother tree with differences in dispersal of its seeds, using pool‐seq (Schlötterer, Tobler, Kofler, & Nolte, 2014) and a whole‐genome genotyping strategy. The goal of our experiment was to determine whether there are loci in the genomes of hybrid chestnut where a *Cd/Cm,* versus a *Cd/Cd,* genotype is associated with greater seed dispersal distance and likelihood of caching versus consumption of nuts. Our research questions were as follows:

1. Do differences in seed size or other heritable characteristics influence differences in the way dispersers handle, consume, and/or cache backcrossed hybrid chestnut seeds?
2. What is the genetic basis of seed traits that lead to differences in seed disperser (squirrel) behavior during interactions with hybrid chestnuts?
3. What models of granivore/seed interaction receive strongest support and what research directions are implied by the data?

## 2 MATERIALS AND METHODS

### 2.1 Seed collection

Seeds were collected in late September and early October from a planting of several hundred BC3 ([(*Castanea mollissima × dentata*)* × dentata*]* × dentata*)* × dentata*) chestnuts at Purdue University's Lugar Farm in Tippecanoe County, IN. The purpose of the planting is blight resistance screening. Most seed parents were 11 years old, but several were 4 years old in 2014 at the start of the study. “Clapper,” a BC1 tree, was the blight‐resistance donor and only source of *Cm* genetic material in this backcross population. *Cm* nuts were obtained from a pair of trees planted as blight‐resistant checks in the Lugar Farm orchards. *Cd* nuts were obtained from two adult trees growing at the Purdue Wildlife Area in Tippecanoe County, IN. BC3 seed parents were chosen based on seed size, with roughly equal numbers of large‐seeded, small‐seeded, and average‐seeded trees chosen, in order to capture a wide range of phenotypic variation. Seed parents were tagged with durable individual plastic nursery labels, but due to large annual variation in the size of seed crops and the loss of some seed parents due to chestnut blight, different seed parents were chosen each year of the study. Seeds were collected by knocking burrs off the parent tree using a ~2‐m wooden pole and manually removing seeds from the bur if necessary. Seeds were floated in water to determine viability; floating seeds were deemed nonviable and discarded.

### 2.2 Seed measurements

Seeds were stored in a cooler (4.4°C) following de‐burring and floating and stratified in peat moss to maintain viability during cold storage. In October, at least 10 seeds from each seed parent were weighed on a digital scale to determine average seed mass. Length (from seed base to tip) and width (across the broadest part of the seed) were determined using digital calipers. In 2015, desiccation was also measured by weighing seeds immediately after collection and again 80 days following collection.

### 2.3 Seed tagging

Tagging was carried out immediately before dispersal trials to avoid spoilage of seeds. A method similar to that employed by Xiao, Jansen, and Zhang (2006) and Hirsch, Kays, and Jansen (2012) was used. A hole was made in the proximal (wider) end of each seed using either a botanical dissecting needle or a small (~2 mm) drill bit. A piece of 24‐gauge green floral wire approximately 12 cm long was looped through the hole and twisted to secure it. A piece of brightly colored waterproof tape was attached to the end of the wire and labeled with a number designating the seed parent.

### 2.4 Dispersal trials

Dispersal trials were conducted in late October, November, and December of each year at four feeding stations placed in and around the Lugar Farm chestnut plantings in Tippecanoe County, IN in a manner similar to the methods of Lichti, Steele, Zhang, and Swihart (2014). In 2016, a feeding station in Woodford County, IL adjacent to the campus of Eureka College was added. At both locations, fruiting chestnuts were present in addition to black walnut (*Juglans nigra*) and several oak species. Fox squirrels (*Sciurus niger*) were the only scatter‐hoarding squirrel species observed at either feeding station. Feeding stations were prebaited to acclimate local squirrels to the feeding locations in August‐September prior to dispersal trials. During dispersal trials, 10 (2016) or 25 (2014–15) seeds from 5 to 6 (2016) or 3 to 4 (2014‐15) parent trees were randomly distributed near a post at the center of each feeding site. Seeds were left out for 4‐5 days, and seed fates (cached, consumed, or left at feeding station) were recorded and dispersal distances measured with a forestry measuring tape attached to the post at the center of the feeding site. Intensive searches for seeds were conducted up to 20 m from the feeding site, although some seeds were found outside this distance due to the high visibility of the tags. Trials started in late October or early November and continued through December until the soil surface froze. Relationships between seed dimensions and dispersal parameters were statistically investigated using the lm and glm packages of R software version 3.2.3 (R Core Team 2015).

### 2.5 DNA Isolation

DNA was isolated from BC3 seed parent trees following dispersal trials. Dormant twigs were collected for DNA extraction in early spring 2016 and 2017. Terminal sections (about 3–5 cm) of first‐year twigs were ground to a fine powder in liquid nitrogen using a mortar and pestle. The ground tissue was placed in 5 ml of heated (50°C) CTAB extraction buffer in a 15‐ml conical tube and incubated 4‐8 hr at 50°C. Following incubation, 1 ml of 20 mg/ml proteinase K solution was added and samples were incubated for an additional 15 min. Five milliliters of 25:24:1 phenol:chloroform solution was added, and samples were purified using a standard phenol:chloroform extraction (Doyle and Doyle 1987) followed by precipitation of DNA using 0.2 M sodium chloride and isopropanol. After pelletting and resuspending samples in TE buffer, contaminants were removed using Zymo Research OneStep PCR Inhibitor Removal kits (Zymo Research). Following purification, samples were quantified using a Nanodrop 8000 (ThermoFisher Scientific), and 2% agarose gel, pooled according to dispersal parameters, then submitted to the Purdue Genomics Core Facility for sequencing.

### 2.6 DNA pooling and sequencing

Pools of samples were made for different phenotypic classes based on (a) mean dispersal distance for cached seeds and (b) frequency of caching. The strong‐dispersal pool (Pool A; eight samples) contained DNA from parents that produced seeds with a long dispersal distance (>5 m average dispersal distance for cached seeds) and high frequency of caching (5%–59%) of recovered seeds in caches), including one Chinese chestnut. A moderate‐dispersal pool (Pool B; seven samples) contained parents that produced seeds with a shorter dispersal distance (<5 m) and low frequency (4%–14%) of caching. The weak‐dispersal pool (Pool C; 10 samples) contained seed parents that produced seeds with a frequency of caching and dispersal distance near 0, including one American chestnut (Table 1). Twenty microliters of DNA at concentration 200 ng/μl from each individual sample was included in a pool, and the combined sample was submitted for library construction and sequencing; samples were sequenced as separate libraries on one Illumina HiSeq 2500 (Illumina Inc., San Diego, CA, USA) lane. Reads were paired‐end, 100 bp in length. The individual genomes of “Clapper,” several unrelated Chinese chestnuts, and two American chestnuts were sequenced separately with two samples per lane as part of a blight resistance study (LaBonte et al., in preparation).

**Table 1:** Summary of seed dispersal data for trees in three genotyping pools, showing the pool each individual parent tree was assigned to, its mean seed weight, the number of seeds cached, the average distance seeds were cached away from the feeding site, and the total number of seeds found for that individual (cached + eaten)

Pool	Species	Year	Mean seed mass (g)	*N* (cached)	Mean distance (m)	Total found	Total offered
A^a^	CC	2014–15	8.53	8	10.49	27	75
A	BC1	2015	3.77	5	8.25	20	25
A	BC1	2015	6.82	13	7.341	21	25
A	BC1	2016	4.14	1	10.98	15	30
A	BC1	2016	3.35	2	7.8	13	30
A	BC1	2014	4.51	7	9.08	19	25
A	BC1	2014	4.03	2	9.64	28	50
A	BC1	2014	2.55	6	6.40	30	50
B^b^	BC1	2014	3.55	3	4.53	23	50
B	BC1	2016	3.16	2	4.92	20	40
B	BC1	2016	4.57	1	4.42	25	50
B	BC1	2016	2.67	1	3.69	23	40
B	BC1	2016	3.50	1	2.85	9	30
B	BC1	2016	3.82	1	3.52	13	30
B	BC1	2016	3.48	1	4.92	9	30
C^c^	BC1	2016	2.53	0	0	12	20
C	BC1	2016	3.22	0	0	9	40
C	BC1	2016	4.01	0	0	24	50
C	BC1	2016	3.66	0	0	14	20
C	BC1	2014	2.99	0	0	24	50
C	BC1	2014	3.47	0	0	24	50
C	BC1	2015	2.54	0	0	19	25
C	BC1	2015	1.47	0	0	7	25
C	BC1	2014	3.09	0	0	24	50
C	AC	2014‐15	1.52	1	1.85	30	50

^a^Strong‐dispersal pool; ^b^Moderate‐dispersal pool; ^c^Weak‐dispersal pool.

### 2.7 Genome assembly and SNP calling

Short reads were assembled to the draft Chinese chestnut reference genome v1.1 (Carlson et al., 2014; Pereira‐Lorenzo et al., 2016) using the Burrows‐Wheeler aligner (bwa) (Li and Durbin 2009). Alignments were processed and polymorphisms called for each pool of samples using Picard Tools and the Genome Analysis ToolKit (GATK) best practices workflow (DePristo et al. 2011; Van der Auwera et al. 2013), minus the quality score recalibration step. When calling SNPs using the HaplotypeCaller tool in the GATK, ploidy was set to twice the number of individuals in the pool.

### 2.8 Analysis of SNP data

Several custom Perl scripts were used for processing of polyploid SNP data files generated by the GATK pipeline. The goal of these scripts was to discriminate between predicted genes that had two genotypes well‐represented in a pool (*Cm/Cd* sites) and genes that had a single genotype fixed or nearly fixed within a pool (*Cd/Cd* sites) for SNPs within the predicted gene sequence, informed by the observation (LaBonte et al. 2018, in preparation) that heterozygosity values in coding sequences of chestnut hybrids are usually much higher than either parent species. There are no *Cm/Cm* sites in a BC3 genome. To identify *Cm/Cd* sites in pooled BC3 genomes, the multi‐sample SNP file was filtered for polymorphisms occurring within predicted genes (AUGUSTUS gene prediction; Stanke et al. 2006) with strong (e‐value <0.001) alignments to the curated Uniprot/SwissProt protein database. For each SNP (minimum depth = 8) between the predicted transcription start and stop sites of each gene, a heuristic hybridity estimator (HE) was assigned to approximate the proportion of individuals that were heterozygotes (*Cm/Cd*) within a pool. If major allele frequency for a SNP within a pool was between 0.45 and 0.55, HE was assigned a value of 0.75 (most individuals heterozygous), if it was between 0.55 and 0.70, HE was 0.5, if it was between 0.70 and 0.85, HE was assigned the value 0.25, and if the major AF was >0.85, HE was assigned 0 (all or nearly all individuals homozygous). This estimate was averaged across all the SNPs in each predicted gene sequence. When more than 50% of the SNPs in a genotype's sequence could not be scored, that genotype‐locus combination was considered missing data. Finally, HE was averaged across 10‐gene bins for each pool and compared, by bin, among pools. Binning was carried out to detect linked *Cm* haplotype blocks and avoid detecting single outlier genes as false‐positives. Loci potentially contributing to interspecific differences in seed dispersal were identified as those 10‐gene bins that had a difference in average HE values >2 standard deviations greater than the average HE difference between the strong‐dispersal pool and the moderate‐ and weak‐dispersal pools. Genes within these regions were annotated using the UniProt entries for aligned proteins from the UniProt KB/ SwissProt database (Xenarios 2016). Predicted molecular interactions were analyzed using the STRING protein database.

All the BC3 trees in our sample inherited 100% of their *Cm* alleles from “Clapper,” but only regions segregating in “Clapper” (loci with *Cd/Cm* genotypes) were informative, that is, about ~50% of the genome of “Clapper” (a BC1 tree), the other half were *Cd/Cd*. Thus, our inference on the genomic basis of interspecific difference in seed dispersal was limited to those heterozygous regions. Of loci that were hybrid in “Clapper,” any given BC3 descendant of “Clapper” was expected to retain a *Cm* allele at one in four loci, with the rest acquiring a second *Cd* allele in two rounds of meiosis. Therefore, at a locus known to have a *Cd/Cm* genotype in “Clapper,” a random sample of “Clapper”‐derived BC3s is expected to have one *Cm* allele observed out of every eight (one *Cm/Cd* and three *Cd/Cd*). As we genotyped BC3s in pools, opportunities for random sampling error were present; a given individual's genotype might be over‐represented at a locus, biasing allele frequency. Over‐representation of an individual could be due to differences in DNA quality, inaccurate estimates of DNA concentration prior to pooling or random inclusion of more DNA fragments from one individual during the high‐throughput sequencing process. We developed a Perl script to estimate the likelihood that more *Cm* alleles than expected by chance alone were present at a given SNP locus in the pooled data.

First, a panel of eight *Cm* genomes with no evidence of hybrid background, two *Cd*, and “Clapper” whole‐genome sequences (LaBonte et al. 2018) were used to filter a the pooled whole‐genome SNP file for loci with one allele fixed in *Cd*, one allele fixed in *Cm*, and a *Cm/Cd* genotype in “Clapper.” The coordinates of these loci were recorded as informative SNPs because markers at those loci allowed us to make inferences about the effects of *Cm* alleles on seed dispersal. Only informative SNPs were kept from the pooled genome SNP file for the analysis.

Next, the program made random draws from arrays of 100 binary values (0 for *Cd*, 1 for *Cm*) set to represent the expected species allele frequency at a given SNP locus for the strong‐dispersal, moderate‐dispersal, and weak‐dispersal pools. As the strong‐dispersal pool contained one *Cm* individual, the expected frequency of *Cm* alleles at a locus that was hybrid in “Clapper” was 3/8 rather than 1/8; therefore, the array of potential alleles contained 38 “1” values and 62 “0” values. The moderate‐dispersal pool only contained BC3s, so 1/8 was the expected fraction of *Cm* alleles. As the weak‐dispersal pool contained one *Cd* individual, the expected fraction of *Cm* alleles was slightly lower (1/10). To simulate the process of pooled DNA assembly, random draws were made from this distribution up to a simulated read depth of 8, and the number of *Cm* alleles in the sample was tallied. This process was repeated 1,000,000 times for each pool to create null distributions for *Cm* allele frequencies in BC3 pooled genomes at “Clapper” hybrid loci and at an assembly depth approximately equal to our actual assemblies.

Subsequently, a *p*‐value was assigned for each informative SNP in a pool, based on the percent of simulated SNP genotypes that had a count of *Cm* alleles greater than or equal to the observed number of *Cm* alleles at that SNP locus. If this percentile‐based *p*‐value was lower than 0.05, the null hypothesis that *Cm* alleles were randomly distributed at the locus in a pool was rejected; low *p*‐values were interpreted as evidence that more *Cm* alleles were present in the pool than expected by chance alone. For each predicted gene in the genome that contained informative SNPs, an average *p*‐value was computed using all informative SNPs within the predicted gene sequence. Predicted genes where the null hypothesis was rejected in the strong‐dispersal, but not in the moderate‐ or weak‐dispersal pools were included as potential candidates for influencing seed dispersal. *p*‐Values assigned to loci using this method were also used to validate candidates identified by the HE heuristic.

### 2.9 Validation of predicted genes

To validate predicted genes from the whole‐genome analysis, cDNA data for a number of species in the order Fagales were aligned to predicted proteins from the *Castanea mollissima* genome (Carlson et al., 2014; Staton et al., 2014; Appendix S1). cDNA contig consensus sequences were aligned to a database of predicted *Castanea* protein sequences using the Diamond sequence aligner (Buchfink et al. 2015). A predicted gene was counted as having transcript support if at least one cDNA contig had the predicted gene's protein sequence as its best alignment. The *Arabidopsis* best hits for each predicted chestnut peptide in genome regions determined to be associated with seed dispersal by the HE method were submitted to gene ontology analysis using g:prolifer (Reimand et al. 2016).

## 3 RESULTS

### 3.1 Seed phenotypes

Dispersal trials were conducted for 13 BC3, one American, and one Chinese chestnut in 2014; 11 BC3, one American, and one Chinese chestnut in 2015; and 12 BC3 in 2016 (Table 1). The average mass (mean ± *SD*) of BC3 seed over the three years was 3.51 ± 1.47 g, ranging between 1.12 and 7.78 g. The average for American chestnut was 3.05 ± 0.17 g; for Chinese chestnut, the average was 7.82 ± 1.01 g. Average seed length of BC3 in 2014 and 2015 was 22.12 ± 2.43 mm, with a range of 17.73–25.86 mm; length for American chestnut was 20.49 ± 0.16, and for Chinese chestnut average length was 24.27 ± 0.69. Width across the wider axis of the attachment‐scar end of the nut was 20.63 ± 3.88 mm for BC3, ranging between 14.42 and 26.4 mm; for American chestnut the mean was 20.04 ± 0.70 mm and for Chinese chestnut 27.29 ± 0.46 mm. In 2015, *Cd* seeds lost more of their mass through drying (15.85%) than *Cm* (10.26%) over 2.5 months of cold‐room storage (October 28 to January 14). The individual half‐sib seed lots with the highest rate of caching (68%, 55%, and 25% of seeds recovered in caches rather than recovered eaten) lost moisture at rates similar to Chinese chestnut (8.99%, 8.55%, and 11.61% of moisture lost, respectively) while seeds that were less likely to be dispersed and cached had highly variable (5.41%–31.64%) loss of mass due to drying and an average rate of moisture loss (17.11%) closer to American than to Chinese chestnut.

### 3.2 Seed dispersal

**Figure 1:**
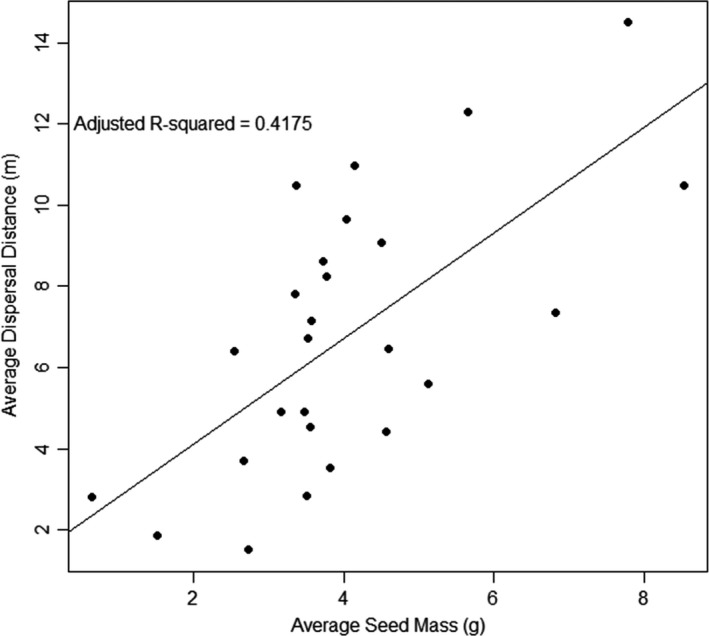
Scatterplot with simple linear regression line of average distance to caching (m) over average seed mass (g) for 25 BC3, 2 *Castanea dentata*, and 2 *C. mollissima* mother trees measured 2014–2016

Average recovery rate (% of tagged seeds recovered after 4–5 days) was 66% in 2014, 42% in 2015, and 46% in 2016. Of seeds that were recovered, in 2014, 36.5% were eaten without being moved away from the feeding site, 49.3% were moved and eaten, and 20.5% were moved and cached. In 2015, these numbers were 40.8%, 36.1%, and 23%, respectively; in 2016, they were 66.1%, 29.0%, and 4.8% cached, respectively. In 2016, the apparent shift in proportions was driven by a low caching rate at the Indiana site rather than the addition of the Illinois site. Average dispersal distance for individual BC3s with more than one dispersal event ranged from 4.92 to 9.08 m, which was less than the average for Chinese chestnut (10.49 m) and greater than the single American chestnut that was cached (1.85 m). Chinese chestnuts were dispersed farther and cached more frequently (29.6% of the time) than American chestnuts (3.2% of the time) and most BC3s (averaged over 14 families with at least one dispersed seed: 16.5%). Pools created for genotyping reflected the wide range of variation in dispersal. The average dispersal distance for seeds of trees placed in the strong‐dispersal pool (seven BC3 and one *Cm*) was 9.08 ± 4.12 m and average caching frequency (no. cached / no. found) 25.3% (Table 1). For the moderate‐dispersal pool (seven BC3), the average distance dispersed was 4.12 m ± 0.78 m, with a caching frequency of 8.2%. In the weak‐dispersal pool (nine BC3 and one *Cd*), the average distance dispersed was 0.185 ± 0.59 m, and the caching frequency was 0.5%. Mean individual seed size was a statistically significant predictor of mean individual caching distance in a simple linear regression where individuals with average seed dispersal distance 0 (i.e., seeds that were only recovered eaten at the feeding platform) were excluded (*t*_1,25_ = 4.43, *p* = 0.0002, adjusted *r* ^2^ = 0.42) and seeds cached/total number of seeds recovered (*t*_1,25_ = 2.26, *p* = 0.03, *r* ^2^ = 0.14) (Figure 1). In a binomial regression, mean seed mass was not a significant predictor of whether an individual mother tree had at least one seed recovered in a cache (*z* value = 1.394, *p* = 0.163).

### 3.3 Pooled genome SNP genotyping

Enough 100‐bp paired‐end reads (57–67 million) were obtained for each pool to cover the ~800 Mb chestnut genome between 7.2 and 8.5 times, so that each individual tree in each pool was represented by about one read at any locus in the genome. A small fraction of total bases (~2%) were removed from each sample by Trimmomatic due to low read quality prior to analysis. In the strong‐dispersal pool, 341363 informative SNPs with coverage >8 were identified; 177,884 were identified in the moderate‐dispersal pool, and 215,590 were identified in the weak‐dispersal pool. As expected, “Clapper” had a *Cd/Cm* genotype at 50% of the loci with one allele fixed in *Cm* and another in *Cd*, and a *Cd/Cd* genotype at the other 50%.

### 3.4 Analysis of hybrid regions among pools

**Figure 2:**
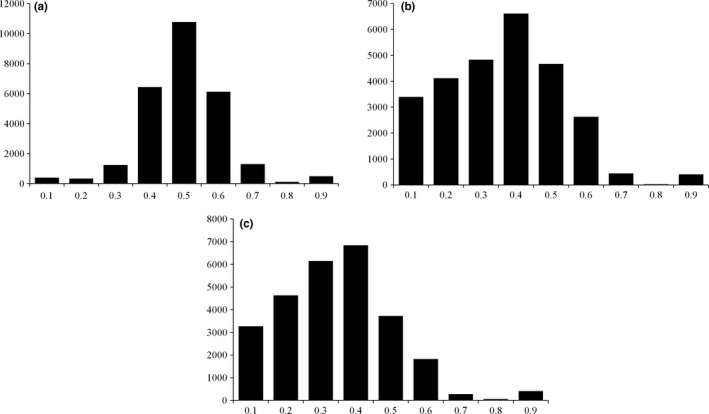
Histograms of heterozygosity estimates for SNPs in the pooled genome sequences of (a) seven BC3 and one Chinese chestnut with more frequent and longer‐distance nut dispersal (b) seven BC3 chestnut with intermediate dispersal distance and low caching frequency and (c) eight BC3 and one American chestnut with low caching frequency and short dispersal distance

The mean value of the heuristic hybridity estimator (HE) over all SNPs in predicted genes with coverage ≥8 was highest for the strong‐dispersal pool (0.44 ± 0.123) (Figure 2) and lowest for the weak‐dispersal (0.294 ± 0.164) (Figure 2). For the moderate‐dispersal pool, the mean value of HE was 0.313 ± 0.174 over all predicted genes (Figure 2). When windows of 10 genes were used, mean difference in HE among windows was greatest between the high‐ and weak‐dispersal pool (0.155 ± 0.088), but the difference between the strong‐ and moderate‐dispersal pools was similar (0.137 ± 0.101) and both were much larger than the average difference between weak‐ and moderate‐dispersal pools (0.019 ± 0.085). Of 2,714 bins of ten predicted genes, there was one region where the difference in HE between the strong‐dispersal pool and the weak‐dispersal pool was >3 standard deviations greater than the mean difference, and 53 bins >2 standard deviations above the mean. There were two bins for which the difference in HE between the strong‐dispersal pool and the moderate‐dispersal pool was >3 *SD* above the mean, and 58 where the difference was >2 *SD* above the mean (Table 2).

**Table 2:** Notable predicted genes within genome intervals identified based on lower major allele fraction (higher proportion of *Cm* alleles) in a pool of BC3 chestnut mother trees with long caching distance and high proportion of seeds cached, relative to other BC3 chestnuts in the study, including predicted molecular function and simulation‐based statistical support for an excess of *Cm* alleles at the predicted gene

LG^a^	BP interval^b^	*SD* c	Gene^d^	Uniprot hit^e^	Annotation^f^	SNPs^g^	*p* < 0.05, pool A^h^	P(A)^i^	P(B)^j^	P(C)^k^	BC1 Het.^l^	*Cd* Het.^m^
LGA	83,772,158–8,408,9674	3.32	A.g10648	LRX3_ARATH (77%)	Extensin‐like protein; cell wall formation	10	2	0.17	0.59	0.59	0.742	0.000
			A.g10657	VIL1_ARATH (48%)	Involved in vernalization, flowering regulation	19	8	0.19	0.87	0.41	0.522	0.042
LGC	48,273,510–48,654,688	3.09	C.g6050	EMF2_ARATH (46%)	Polycomb group protein; flower development	26	8	0.24	0.37	0.82	0.838	0.068
LGG	40,408,926–40,687,727	2.61	G.g5214	DLO2_ARATH (39%)	Salicylic acid catabolic process	6	4	0.06	0.91	0.03	0.446	0.175
LGH	46,084,023–46,409,469	2.29	H.g5097	LRP1_ARATH (50%)	Involved in formation of female flower parts	9	2	0.38	0.84	0.82	0.093	0.018
LGI	25,672,061–25,978,423	2.71	I.g3291	NLTL5_ARATH (33%)	Lipid binding and transport	1	0	0.05	0.00	na	0.429	0.000
			I.g3304	C94A2_VICSA (52%)	Hydroxylation of fatty acids	1	0	0.64	0.91	0.64	0.600	0.000
LGL	59,955,058‐60,179,331	2.92	L.g7556	PME51_ARATH (59%)	Demethylesterification of cell wall pectin	2	0	0.05	0.91	na	0.160	0.031
			L.g7302	CESA2_ARATH (78%)	Crystallization of cell wall microfibrils	10	0	0.48	0.91	0.86	0.203	0.092
LGL	65,537,114‐65,794,683	2.89	L.g8192	TPS13_RICCO (62%)	Sesquiterpene synthase	50	10	0.18	0.91	0.42	0.700	0.022
			L.g8191	NES1_FRAAN (61%)	Synthesis of volatile mono‐, sesquiterpenes	3	1	0.43	0.91	0.35	0.671	0.155
			L.g8198	NES1_FRAVE (58%)	Synthesis of volatile mono‐, sesquiterpenes	31	3	0.39	0.91	0.27	0.554	0.142
			L.g8208	NES2_FRAAN (60%)	Synthesis of volatile mono‐, sesquiterpenes	9	2	0.30	0.91	0.42	0.667	0.123

^a^Linkage group and ^b^base position in the Chinese chestnut draft pseudochromosome assembly (Carlson et al., 2014); ^c^Number of standard deviations that the heterozygosity estimator (HE) for the strong‐dispersal pool exceeded either the weak‐dispersal or no‐dispersal pools; ^d^Numeric code for a predicted gene (AUGUSTUS) in the Chinese chestnut draft reference genome (Carlson et al., 2014); ^e^Top hit from the Uniprot/Swissprot curated protein sequence database for the predicted chestnut gene, with percent amino acid identity; ^f^Likely function based on the top Uniprot hit; ^g^Informative SNPs in the gene sequence; ^h^SNPs where *Cm* allele frequency in the strong‐dispersal‐pool was in the top 5% of simulated random *Cm* allele frequencies; ^i^Average percentile value of all SNPs (smaller value = more *Cm* alleles than expected) in the predicted gene for the strong‐dispersal pool; ^j^Average percentile value of SNPs in the weak‐dispersal pool; ^k^Average percentile value of SNPs in the no‐dispersal pool; ^l^Heterozygosity for all SNPs in the gene for “Clapper,” the *Cm* allele donor for all BC3 trees in the experiment; ^m^Heterozgosity for all SNPs in the gene, averaged over two individual *Cd* samples.

### 3.5 Annotations of genes within hybrid regions

**Figure 3:**
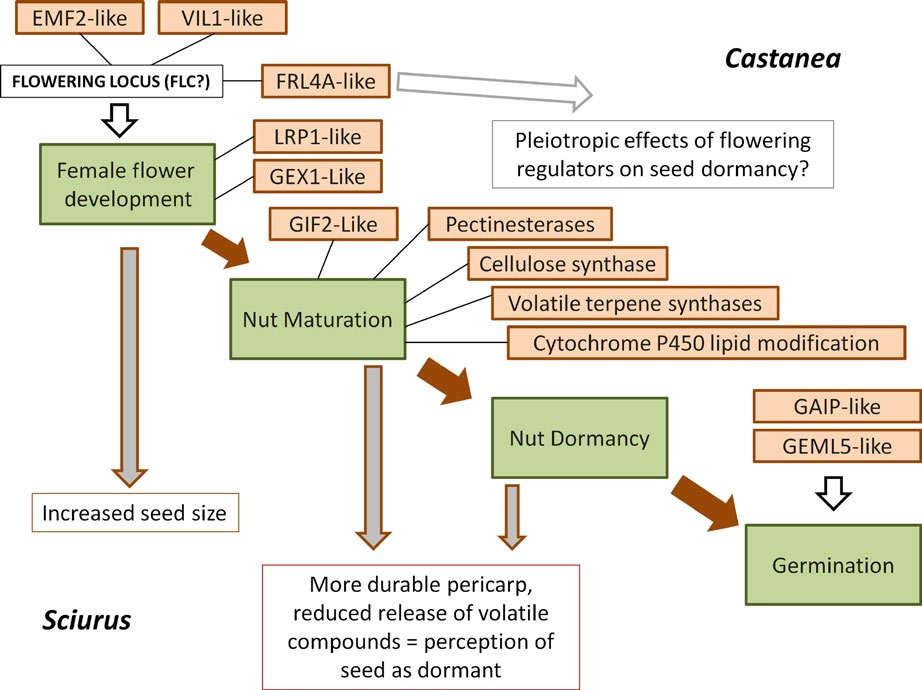
Depiction of the role of candidate genes in *Castanea* nut development (upper right) and the perceptions of nut phenotypes by squirrels (Sciurus) that are hypothesized to cause some BC3 chestnuts to be dispersed farther and cached more frequently than others

Candidate genes for differences in seed dispersal were analyzed for 18 bins with the largest deviations from the mean difference in the heterozygosity estimate between the strong‐dispersal pool and the moderate‐ and weak‐dispersal pools. Fourteen of these bins had a large difference in heterozygosity between the strong‐dispersal pool (pool A) and the others (pools B and C); three were identified based on the difference between the strong‐ and moderate‐dispersal pool; and one was identified based on the difference between strong‐ and weak‐dispersal pools while the strong and moderate‐dispersal pools showed no difference (Table 2). Of these 14 genome regions, seven that had additional support from the simulation‐based estimation of significance were chosen as the most likely candidates (Table 2). Additional individual candidate genes (rather than regions) were identified based on simulation‐based evidence of *Cm* alleles in the strong‐dispersal pool (Table 3). Examining annotations of predicted genes in these regions revealed several that have plausible roles in seed development and subsequent seed handling and dispersal by squirrels (Figure 3). Many of the predicted genes in these regions aligned to cDNA sequences from chestnuts and other nut‐bearing species in the order Fagales (Table 5). Gene ontology terms that were enriched in candidate regions, as determined by the difference in HE between strong‐dispersal and weak‐dispersal pools, included “regulation of cellular localization” (*p* = 0.0181), “membrane‐bounded organelle” (*p* = 0.0428), and “plasma membrane” (*p* = 0.0181).

**Table 3:** Individual predicted genes identified as candidates for differences in seed dispersal based on significant departures from expected allele frequencies in a pool of BC3 chestnut mother trees with long caching distance and high proportion of seeds cached, relative to other BC3 chestnuts in the study

Gene^a^	Strong‐dispersal SNPsA^b^	Significant SNPs^c^	*F*_ST_ d	Uniprot^e^	Annotation
A.g11556	9	0.82	0.343	PME_PRUPE	Pectin methylesterase; cell wall remodeling
A.g1855	6	0.86	0.349	GWD2_ARATH	Alpha‐glucan water dikinase
A.g1991	5	1.00	0.655	BH030_ARATH	bHLH transcription factor
A.g3246	5	1.00	0.731	C94A2_VICSA	Cytochrome p450 oxidase; fatty acid hydrolysis
A.g5184	3	1.00	0.811	BZR1_ARATH	Modulates ovule development; brassinosteroid signaling
A.g5562	8	0.89	0.319	GEX1_ARATH	Gametophyte development and embyrogenesis
A.g6140	7	0.78	0.965	AOP1C_ARATH	Dioxygenase potentially involved in producing glucosinolates
A.g7734	1	1.00	0.564	GEML5_ARATH	Maintenance of seed dormancy by abscisic acid
A.g8091	5	0.83	0.496	LHT1_ARATH	Lysine histidine amino acid transporter
B.g3059	4	0.80	0.252	PERR_RAUSE	Biosynthesis of monoterpenoid indole alkaloids
E.g1046	11	0.92	0.662	C76AD_BETVU	Cytochrome p450 oxidase in the betalain synthesis pathway
E.g4071	14	0.78	0.240	WAK4_ARATH	Cell‐surface kinase that binds to pectin; cell expansion
E.g7467	3	1.00	0.589	STC_RICCO	Sugar carrier protein C; carbohydrate transport
E.g8229	8	0.80	0.899	HPPR_PLESU	Hydroxyphenylpyruvate reductase; Biosynthesis of rosmarinic acid
E.g911	5	0.83	0.719	GAIP_CUCMA	DELLA protein; repressor of gibberellin signal pathway
F.g2981	21	0.81	0.865	TRA1_MAIZE	Putative AC transposase from transposon “Activator”
F.g927	4	1.00	0.365	RKS1_ARATH	Carbohydrate‐binding serine/threonine protein kinase
F.g941	10	0.77	0.885	SD16_ARATH	Receptor‐like kinase; regulation of cellular expansion
H.g315	6	0.86	0.433	BPG2_ARATH	Brassinosteroid‐mediated transcriptional regulation
J.g6035	4	1.00	0.583	AKR1_SOYBN	Aldo‐keto reductase
K.g1622	3	1.00	0.749	BIOF_ARATH	8‐amino‐7‐oxononoanoate synthase
K.g2221	4	0.80	0.565	FRL4A_ARATH	FRIGIDA‐like protein; flower development
L.g6163	4	0.80	0.910	MTEF8_ARATH	Transcription termination factor
L.g6184	13	0.77	0.722	GIF2_ARATH	Transcription coactivator; active in cotyledon tissue

^a^AUGUSTUS predicted gene from the Chinese chestnut draft reference genome (Carlson et al., 2014) preceded by linkage group letter; the initial letter indicates the pseudochromosome in which the predicted gene was found; ^b^SNPs in the upper 5% of the distribution of simulated random *Cm* allele frequencies in the strong‐dispersal pooled genome. No SNPs significant at this level were identified in these intervals in the weak‐ or no‐dispersal pools; ^c^Percent of informative SNPs in the gene sequence, for the high‐dispersal pool, in the upper 5% of simulated *Cm* allele frequencies; ^d^ *F*_ST_ calculated for SNPs within the predicted gene among a sample of individual Chinese and American chestnut genome sequences; ^e^Top hit for the predicted protein from the Uniprot/Swissprot curated protein database.

## 4 DISCUSSION

### 4.1 Dispersal trials

Previous studies have indicated that seed‐caching rodents are more likely to cache (rather than eat) relatively large seeds, and carry larger seeds farther before caching (Jansen et al., 2002; Moore et al., 2007; Tamura & Hayashi, 2008; Xiao et al., 2005). The dormancy status of seeds is also likely a factor in caching decisions (Moore et al., 2007; Smallwood et al., 2001; Xiao, Gao, Jiang, & Zhang, 2009; Xiao, Gao, Steele, & Zhang, 2009): squirrels are more likely to eat seeds perceived as nearing germination and to cache seeds perceived as dormant. The value of squirrel caches to nut survival (fitness) has been amply demonstrated (Lichti, Steele, & Swihart, 2017), so seed phenotypes that influence squirrel caching have evolutionary significance for nut‐bearing trees. In our study, seed size, as measured by seed mass, was associated with both dispersal distance (Figure 1) and likelihood of caching vs. consumption. Seed mass was highly variable among the backcrossed trees in the study, which we deliberately chose based on seed size (Table 1) from a much larger population of BC3 chestnuts. Seeds with greater mass tended to exhibit greater dispersal distance and greater likelihood of caching vs. immediate consumption by squirrels. Chinese chestnuts in this study were much larger than American chestnuts (Table 1), so BC3 hybrids with larger seeds (similar to Chinese chestnut) were more likely to be cached. Variation in seed dispersal distance and caching likelihood among BC3 trees, however, was not fully explained by seed size (Figure 1). There is no documented difference in seed dormancy between American and Chinese chestnut—both species must undergo a dormant period of several months to germinate (Saielli et al. 2012), and in both species, the seed is metabolically active during its dormant phase because chestnuts are recalcitrant seeds (Leprince, Buitnik, & Hoekstra, 1999; Roach et al., 2009). The sugar content of chestnuts under cold storage increases while starches diminish (Ertan, Erdal, Gulsum, & Algul, 2015). Differences in genes that regulate dormancy or signals that communicate dormancy to squirrels, or binding sites for regulatory molecules, are therefore less likely to be false‐positives than structural or housekeeping genes.

### 4.2 Genome scan for loci involved in caching decisions

Interspecific hybrid phenotypes are not always intermediate between parents (Woeste et al. 1998), and intermediate genotypes do not simply correspond to intermediate phenotypes. In hybrid chestnuts, for example, gene interactions can cause unpredictable dormancy‐related phenotypes: hybrid seeds derived from a cross with Allegheny chinkapin (*Castanea pumila*) as pollen parent and *Cm* as the seed parent exhibited reduced seed dormancy (Jaynes 1963; Metaxas 2013). No precocious germination of seeds was observed in the course of our experiment, but subtle phenotypes may have been present. In red oaks (*Quercus* section Lobatae), which are closely related to chestnuts, dormancy and germination appear to be controlled primarily by the pericarp, the dry fruit structure that makes up the hard outer “shell” of both oak acorns and chestnuts (Smallwood et al., 2001). There is evidence that squirrels make use of changes in the pericarp to sense impending germination in oaks and chestnuts. These changes include degradation of pericarp waxes and the release of low molecular weight volatile compounds from inside the pericarp (Paulsen et al., 2014; Sundaram et al. 2015, Sundaram, 2016). By demonstrating that a germinating white oak embryo inside a “dormant” red oak shell is perceived as dormant by squirrels, Steele et al. (2001) showed that signals at the pericarp surface may be more important than signals from the kernel. If some chestnut hybrids have a thicker pericarp wax layer than American chestnut, squirrels might perceive the seeds as reliably dormant and cache them more frequently.

By sequencing pools of chestnuts with different seed dispersal phenotypes, we attempted to identify regions of the genomes of BC3 hybrids where Chinese chestnut allele frequencies were higher than expected in the most frequently dispersed trees. The HE statistic seems to have captured the elevated heterozygosity that is characteristic of *Cm/Cd* hybrid gene loci. The inference space for our study was limited to the phenotypic effects, in a BC3 population, of loci where “Clapper” may have contributed a *Cm* allele, which only included half of the genome. Our ability to find genomic regions that were hybrid in one pool but not the others was impeded by the uncertainty associated with estimating heterozygosity in pooled sequence data. By comparing results from pooled data with individual chestnut genome sequences, we determined that the individual “Clapper” genome was significantly more heterozygous than individual *Cd* genomes at SNP loci in most of the seed dispersal candidate regions (Table 4), which indicated that these regions were plausible candidates. Differences in *Cm* allele frequencies among pools (Figure 2) were accounted for by the simulation‐based method for identifying outliers; only a small fraction of the many genes with divergent allele frequencies between *Cd* and *Cm* were included in candidate regions. The study design limited our inference to maternal effects, but as squirrel caching decisions appear to be influenced strongly by characteristics of the maternally derived pericarp (Steele et al. 2001), the paternal contribution to differences in dispersal is likely to be small. Finally, the small number of genotypes utilized (three pools derived from 24 individual trees) limits the strength of conclusions drawn from this study because the number of false‐positive candidate loci is inversely related to sample size. The importance of candidate genes that remained after statistical validation was rendered more plausible, however, because their predicted function often corresponded to factors known to influence squirrel caching decisions—seed size and the perception of dormancy. These remaining candidates point to new hypotheses on seed/seed‐disperser coevolution in hardwood trees. Seed size, the most obvious dispersal‐associated phenotype that distinguishes *Cd* and *Cm*, is a likely a complex trait in chestnut as it is in other plants (e.g., Gnan, Priest, & Kover, 2014). Several candidate loci identified in this study had annotations that point to a potential role in seed development and seed size. The EMBRYONIC FLOWER 2‐like (EMF) gene on LGC (c.g6050; Table 2) could directly influence seed size by regulation of development of female flower parts. EMF2 in *Arabidopsis* encodes a Polycomb group protein (Yoshida et al., 2001) that regulates vegetative growth and development by suppressing the flower‐development program, Yoshida et al., 2001). The predicted EMF2 gene in chestnut had strong transcript support from *C. mollissima* and *C. dentata* and was one of 5 EMF2‐like genes predicted (by AUGUSTUS) in the entire chestnut genome (Carlson et al., 2014). A candidate gene on LGL (g6184) was similar to GIF2 of *Arabidopsis*, which regulates the expansion of cotyledons (Kim & Kende, 2004). Cotyledons make up the majority of the mass of a chestnut seed. Several other candidates, including a LATERAL ROOT PRIMORDIUM 1 (Kuusk, Sohlberg, Magnus, & Sundberg, 2006) homolog on LGH, a VERNALIZATION‐3‐like gene (VIL1) on LGA from (Table 2), a gene similar to FRIGIDA‐like 4 of *Arabidopsis* on LGK, also may function in the regulation of flower development. The latter two loci are both involved in the FLOWERING LOCUS C (FLC) regulatory pathway in Arabidopsis (Greb et al., 2007; Michaels, Bezerra, & Amasino, 2004). Whether homologs of these flowering regulatory loci have effects on the development of floral parts, and thereby influence seed size in chestnut, is uncertain. It is also possible that they affect dispersal by modifying seed dormancy, given that FLC and its interactors have documented pleiotropic effects on the regulation of seed dormancy and germination (Chiang et al. 2009). If such pleiotropic effects exist, changes in seed size due to natural selection could also lead to changes in dormancy.

**Table 4:** Selected candidate genes with summary of evidence for involvement in seed dispersal. Boldface indicates candidate genes with the strongest evidence

Site	Gene	Pool A^a^	Pool B^b^	Pool C^c^	Het.^d^ BC1	Het.^e^ *Cd*	UniProt^g^	% ID
Sd01	A.g4714	0.488	0.026	0.067	0.120	0.013	DBR_TOBAC	64
Sd02	A.g8635	0.750	0.088	0.242	0.176	0.062	ASR1_SOLLC	82
**Sd03**	**A.g10648**	**0.650**	**0.145**	**0.206**	**0.742**	**0.000**	**LRX3_ARATH**	**77**
**Sd03**	**A.g10657**	**0.522**	**0.077**	**0.195**	**0.522**	**0.042**	**VIL1_ARATH**	**48**
Sd04	A.g13357	0.585	0.124	0.154	0.026	0.055	SEC5A_ARATH	57
Sd04	A.g13359	0.471	0.104	0.063	0.007	0.043	SEC5B_ARATH	71
Sd05	B.g1118	0.470	0.093	0.082	0.108	0.035	GOLS2_ARATH	72
Sd06	B.g3452	0.422	0.089	0.119	0.119	0.021	SUP_ARATH	41
Sd06	B.g3458	0.534	0.031	0.180	0.305	0.088	CSLG2_ARATH	36
Sd06	B.g3460	0.522	0.000	0.174	0.227	0.068	CSLG2_ARATH	38
Sd07	B.g5164	0.449	0.289	0.260	0.459	0.183	UXS2_ARATH	70
Sd08	C.g3074	0.437	0.077	0.093	0.236	0.042	PME31_ARATH	79
Sd09	C.g3725	0.432	0.399	0.062	0.755	0.008	AAPT1_ARATH	85
Sd09	C.g3728	0.516	0.287	0.061	0.787	0.025	AAPT1_ARATH	98
Sd10	C. g6050	0.583	0.325	0.155	0.838	0.068	EMF2_ARATH	46
Sd12	G.g4366	0.175	0.000	0.483	0.857	0.033	ABR1_ARATH	55
Sd12	G.g4369	0.484	0.012	0.369	0.55	0.130	AMYA_VIGMU	71
Sd12	G.g4370	0.460	0.018	0.434	0.592	0.198	LAG12_ARATH	66
**Sd13**	**G.g5214**	**0.557**	**0.066**	**0.580**	**0.446**	**0.175**	**DLO2_ARATH**	**39**
Sd14	H.g5907	0.647	0.078	0.072	0.093	0.018	LRP1_ARATH	50
**Sd15**	**I. g3291**	**0.730**	**0.000**	**0.070**	**0.429**	**0.000**	**NLTL5_ARATH**	**33**
Sd15	I.g3304	0.550	0.000	0.082	0.600	0.000	C94A2_VICSA	52
Sd16	L.g7302	0.535	0.066	0.108	0.203	0.092	CESA2_ARATH	78
**Sd17**	**L.g7556**	**0.517**	**0.050**	**0.129**	**0.160**	**0.031**	**PME51_ARATH**	**59**
Sd18	L.g8191	0.516	0.236	0.419	0.671	0.155	NES1_FRAAN	61
**Sd18**	**L.g8192**	**0.597**	**0.112**	**0.316**	**0.700**	**0.022**	**TPS13_RICCO**	**62**
Sd18	L.g8198	0.667	0.098	0.429	0.554	0.142	NES1_FRAVE	58
Sd18	L.g8208	0.527	0.078	0.455	0.667	0.123	NES2_FRAAN	60

^a^Pool of individuals with highest mean dispersal distance and largest % of seeds cached rather than consumed; ^b^Pool of individuals with lower mean dispersal distance and lower caching %; ^c^Pool of seeds that were rarely or never cached; ^d^Proportion of heterozygous snps in gene for “Clapper” calculated using Vcftools; ^e^Proportion of heterozygous snps in gene for *Cd* calculated in Vcftools; ^f^Percentile of expected *Cm* allele frequency distribution based on 1,000,000 simulated pooled genotypes for each pool, averaged over all SNP loci within gene.

### 4.3 Genomic loci associated with differences in seed dispersal: pericarp‐mediated dormancy

In red oak acorns, which are anatomically similar to chestnuts, the pericarp prevents absorption of water by the embryo and allows germination only after the pericarp's permeability has increased following a period of cold storage (Peterson 1983; Steele et al., 2001). The pericarp is derived from the ovary walls of female chestnut flowers and consists of several layers of lignified cells with a waxy coating on the outermost layer. Both the breakdown of this waxy layer and the subsequent release of volatile compounds from the pericarp may serve as olfactory cues for squirrels that a seed is approaching germination and is therefore more perishable and a poor candidate for caching. The candidate genes we identified include some that may be involved in the formation of pericarp cells, some that may influence the composition of waxes on the pericarp surface, and others that may influence the release of volatile compounds from the pericarp. While none of the transcriptomic data from Fagales trees we aligned to the *Cm* predicted gene set was seed specific, several dispersal candidate genes were supported by cDNA contigs from several species (Table 5).

**Table 5:** Transcriptome alignments from members of the order Fagales, for selected predicted genes from chestnut genome regions associated with interspecific differences in seed dispersal

Gene	Uniprot	*Cm* a	*Cd* b	*Cc* c	*Cs* d	*Qa* e	*Qf* c	*F* g	*Jr* h	*Jn* i	*Ca* j	*Aru* k	*Arh* l	*Bet* m
A.g1855	GWD2_ARATH	100^n^	100	94.5	98.7	100	97.9	–o	77.5	83.6	–	–	–	–
A.g1991	BH030_ARATH	100	–	–	–	–	–	–	62.3	65.3	–	–	–	58.3
A.g3246	C94A2_VICSA	98.7	97.3	87.2	89.2	87.6	95.7	–	–	–	–	–	–	–
A.g5184	BZR1_ARATH	100	100	80.3	81.5	97.4	81.0	–	75.0	75.6	–	90.2	79.7	74.0
A.g7734	GEML5_ARATH	69.4	60.6	99.6	99.4	–	94.2	–	68.0	70.3	90.6	84.4	70.6	83.6
A.g8091	LHT1_ARATH	95.5	100	–	–	–	–	78.1	–	–	–	–	–	–
A.g10648	LRX3_ARATH	100	100	97.9	98.2	93.1	95.1	95.5	–	94.3	95.7	94.3	92.5	90.3
A.g10657	VIL1_ARATH	100	100	100	93.1	96.1	96.2	–	83.0	83.0	84.4	92.4	89.0	87.9
A.g11556	PME_PRUPE	100	–	–	–	–	87.7	–	78.5	–	84.6	–	72.4	76.8
C.g6050	EMF2_ARATH	98.9	98.4	–	–	–	–	–	–	–	–	–	–	74.3
E.g7467	STC_RICCO	94.8	86.8	88.1	91.6	78.6	85.9	77.5	–	–	–	–	–	81.2
E.g8229	HPPR_PLESU	97.9	–	–	–	–	82.3	–	72.3	79.2	81.8	–	84.3	78.1
F.g927	RKS1_ARATH	100	84.7	96.0	92.6	–	75.8	77.1	68.4	–	–	–	–	72.2
G.g5214	DLO2_ARATH	100	99.4	–	–	–	72.5	–	–	–	–	–	–	–
H.g315	BPG2_ARATH	89.6	94.0	–	–	–	81.5	94.5	81.1	80.7	–	–	85.1	85.6
H.g5907	LRP1_ARATH	79.4	100	80.2	69.6	100	88.9	–	58.3	63.0	–	–	–	82.6
I.g3291	NLTL5_ARATH	89.0	100.0	85.0	–	–	77.3	–	50.3	65.1	61.8	–	–	59.8
J.g6035	AKR_SOYBN	100	84.9	–	72.3	–	–	88.2	–	86.4	–	–	–	80.7
K.g2221	FRL4A_ARATH	81.9	100	85.5	86.1	100	84.5	79.7	67.9	65.0	84.6	97.3	70.2	78.4
L.g6163	MTEF8_ARATH	100	–	–	–	–	100	–	72.6	72.5	68.9	–	–	85.9
L.g6184	GIF2_ARATH	93.0	–	–	–	74.5	98.0	–	40.2	57.1	–	70.8	49.1	76.7
L.g7302	CESA2_ARATH	85.6	100	90.0	88.3	100.0	87.3	86.0	84.4	88.5	100.0	79.1	60.5	81.9
L.g8191	NES1_FRAAN	93.1	–	–	–	–	–	–	–	38.9	–	–	–	–
L.g8192	TPS_RICCO	–	–	–	–	100.0	–	–	–	–	–	–	–	–
L.g8198	NES1_FRAVE	–	92.1	–	–	–	95.7	–	–	–	–	–	–	–
L.g8208	NES2_FRAAN	–	100	–	–	–	90.8	–	68.2	81.2	81.2	–	–	78.8

^a^Healthy stems, blight cankers, and whole‐plant tissues (Barakat et al. 2009, 2012) for Chinese chestnut, *Castanea mollissima* (*Cm*) and ^b^American chestnut, *C. denata* (*Cd*); ^c^Root transcriptomes for Japanese chestnut, *C. crenata* (Cc), and ^d^European chestnut, *C. sativa* (Cs) (Serrazina et al. 2015); Whole‐plant libraries of ^e^white oak (*Quercus alba*;* Qa*), ^f^northern red oak (*Quercus rubra; Qr*), ^g^American beech (*Fagus grandifolia; Fg*), ^h^black walnut (*Juglans nigra; Jn*), ^i^red alder (*Alnus rubra; Aru*), and ^j^white alder (*Alnus rhomboids; Arh*) downloaded from hardwoodgenomics.org; ^k^Persian walnut (*Juglans regia; Jr*) whole‐plant transcriptome data (Martinez‐Garcia et al., 2016); ^l^Common hazelnut (*Corylus avellana; Ca*) all‐tissue library (Rowley et al., 2012); ^m^ *Betula platyphylla* (Mu et al., 2012); ^n^Percent amino‐acid identity in a blastx alignment of the predicted chestnut protein with the cDNA transcript; ^o^Predicted protein in chestnut was not the best blastx alignment for any transcript.

Our analysis identified several predicted genes that may have a role in cell wall modification during nut development and ripening in chestnut. These include an extensin‐like predicted gene on LGA (LGA.sd03; g10648), which has a similar protein sequence to an LRX‐family gene in *Arabidopsis* (Baumberger et al., 2003); the LRX family has been implicated in the modification of plant cell walls (Draeger et al., 2015). Predicted genes similar to pectin methylesterases were identified on LGA (g11556) and LGL (g7556). The latter predicted gene (LGL.Sd17) was similar to a gene in *Arabidopsis* expressed in developing siliques (seed pods) (Louvet et al., 2006) and could be involved in the formation of the lignified chestnut pericarp. Pecinesterase genes are active in maturing (lignifying) wood in poplar (Mellerowicz, Baucher, Sundberg, & Boerjan, 2001). Conversely, these genes could be directly involved in the germination process: in yellow cedar (*Chamaecyparis nootkaensis*), pectinesterases are active in germinating seeds (Ren & Kermode, 2000), and *Arabidopsis* lines with overexpression of a PME inhibitor showed more rapid germination (Müller et al. 2005). Given the importance of the pericarp in squirrel perceptions of seed perishability, these cell‐wall modification genes may influence seed dispersal by acting in the maturing pericarp, rather than in the germinating embryo. Other candidate genes that could influence formation of the pericarp include a cellulose synthase (CESA2)—like gene (Beeckman et al., 2002; Li et al. 2009). Adjacent to the pectinesterase at SD17 on LGL and a gene similar to a sugar transport carrier in castor bean (*Ricinus communis*) (STC_RICCO; LGE_g7467). Several candidate genes we identified appear to have a role in lipid metabolism that may be related to the formation and/or degradation of pericarp wax layers (Pollard, Beisson, Li, & Ohlrogge, 2008). The potential importance of fatty‐acid metabolic processes in regulating squirrel dispersal was explored by Sundaram (2016), who showed that differences in the outer wax layer of the pericarp influence squirrel perception of seed dormancy. Nonspecific lipid transfer proteins (LGC.Sd15) in *Arabidopsis* are involved in the formation of suberin in crown galls (Deeken et al., 2016), various tissues of tomato in response to drought stress (Trevino & O'Connell, 1998), and the surface wax of broccoli leaves (Pyee, Yu, & Kolattukudy, 1994). Another lipid‐modifying gene, a cytochrome p450 oxidase (g3304), occurs at the same locus as the NLTL‐like predicted gene on LGC, and a second cytochrome p450 oxidase (LGA_g3246) was identified as a candidate gene on LGA. Based on alignments to the UniProt database, these genes are both similar to a gene from tare (*Vicia sativa*) that catalyzes hydroxylation of myristic acid and other fatty acids (Le Bouquin, Pinot, Benveniste, Salaun, & Durst, 1999).

Squirrels perceive volatile compounds from seeds as cues of metabolic activity and impending germination (Sundaram, 2016). Volatile compounds are thought to escape the pericarp as it becomes more porous and germination approaches. One particularly interesting locus appeared to contain a cluster of four volatile terpene synthase genes, which are most similar to terpene synthesis genes highly expressed in the fruits of strawberry (Aharoni et al., 2004) that are thought to influence the fruit's flavor and aroma profile. Nerolidol is a sesquiterpene compound found in many plants (Chan, Tan, Chan, Lee, & Goh, 2016). Sundaram (2016) found that release of beta‐amyrin, a triterpene, was associated with germination of chestnuts. While these compounds are distantly related, their synthesis may be metabolically linked by production of the intermediate squalene. Nerolidol synthase converts farnesyl diphosphate (FPP) to nerolidol. In a yeast study, overexpressing FPP synthase and squalene synthase greatly increased beta‐amyrin production (Zhang et al., 2015). Beta‐amyrin has been associated with wax degradation in other plants (Buschhaus & Jetter, 2012), so it could degrade the cuticular waxes of the outer pericarp as it is released, preparing the seed for germination (Sundaram, 2016). The genes found here do not directly influence beta‐amyrin production, but could influence production of substrate molecules or divert carbon away from beta‐amyrin production. Interestingly, of the three nerolidol synthase‐like genes at this locus, only one showed evidence of expression in Chinese chestnut and two others showed evidence of expression in American chestnut and oaks, but not Chinese chestnut (Table 4), possibly indicating interspecific differences in the expression of these genes. There was little evidence of their expression in the non animal‐dispersed taxa examined (*Alnus*,* Betula*) nor in *Fagus*. If the expression of multiple copies of nerolidol synthase in American chestnut leads to an increase in the activity of volatile organic compounds that degrade pericarp waxes, the result in BC3 seeds that express the *Cd* alleles could be a signal to squirrels to eat rather than cache these seeds. Our results support previous studies (e.g., Smallwood et al., 2001) that point to seed dormancy and germination as a primary influence on squirrel caching decisions. In particular, they support the notion that pericarp waxes and volatile compounds are important for conditioning squirrels’ perceptions of seed dormancy in nut‐bearing trees. The potential evolutionary role of loci with pleiotropic effects on flower development, seed size, and germination in nut‐bearing trees merits further study. The possibility that lipid‐ and secondary metabolite‐synthesis genes expressed in developing pericarp tissues are ultimately important for seed dispersal phenotypes should be investigated in squirrel‐dispersed tree species and interspecific hybrids. The results of the present study need to be validated and clarified using larger numbers of plants and individual, rather than pooled, genotypes. We hypothesize that genes controlling differences in seed dispersal are primarily expressed during flower and seed development and maturation (the formation of the pericarp) rather than during dormancy, when dispersal takes place.

## 5 CONCLUSIONS

The ecological relationship between trees in Fagales and the scatter‐hoarding rodents and birds that disperse and consume their seeds is pivotal for the current canopy composition and future trajectory of forest ecosystems throughout the northern temperate and subtropical zones. As caching decisions made by squirrels determine whether or not a given seed has a chance of germinating and reproducing, the basis of these decisions has likely been a factor in the evolution and diversification of nut‐bearing tree lineages. Our work provides additional evidence that pericarp‐mediated dormancy plays a predominant role in influencing squirrel dispersal of seeds of the same or closely‐related species (Smallwood et al., 2001; Steele et al., 2001) and the first evidence of gene loci under selection in the coevolution of hardwood trees with scatter‐hoarding seed dispersers. The interplay between differences in seed size, seed dormancy, and the role these traits have played in the evolution, diversification, and speciation of nut‐bearing hardwood trees should be further investigated using more robust genome‐scale genotyping and additional interspecific hybrids. Given the evidence for expression of many of the candidate loci in trees in the order Fagales, it is possible that the predicted genes identified here have had a role in diversification and speciation of several nut‐producing lineages. Additional screening of these candidate genes in the chestnut, oak, and other animal‐dispersed Fagales lineages should further elucidate their role in the ecological coevolution of hardwood trees and their coevolved conditional mutualist seed dispersers.

## CONFLICT OF INTEREST

None declared.

## AUTHOR CONTRIBUTIONS

Dr. Woeste contributed study design and the core questions of the study. Dr. LaBonte carried out seed‐dispersal trials, seed measurements, genotyping, and data analysis.

## DATA ACCESSIBILITY

Quality‐filtered Illumina whole‐genome sequencing reads are available as a Sequence Read Archive (https://www.ncbi.nlm.nih.gov/sra) at the addresshttps://www.ncbi.nlm.nih.gov/bioproject/PRJNA474773). Chinese chestnut genome scaffold sequences and predicted proteins are available at hardwoodgenomics.org.
