# Supplementary material for: Pooled whole‐genome sequencing of interspecific chestnut (Castanea) hybrids reveals loci associated with differences in caching behavior of fox squirrels (Sciurus niger L.)
Source: Ecol Evol. 2018 Oct 23;8(22):10638–54. doi: 10.1002/ece3.4336 (PMC6262733; doi:10.1002/ece3.4336)
Supplement: Supplementary file 1 [file ECE3-8-10638-s001.docx]

Appendix 1. Sources of transcriptomic data from the order Fagales.

| **Species** | **Tissue** | **Reference** | **Website** |
| --- | --- | --- | --- |
| *Castanea mollissima* | Stem, some flowers and roots | Barakat et al. 2009, 2012 | http://hardwoodgenomics.org/transcriptomes |
| *Castanea dentata* | Stem with some flowers and roots | Barakat et al. 2009, 2012 | http://hardwoodgenomics.org/transcriptomes |
| *Castanea sativa* | Roots | Serrazina et al. 2015 | http://hardwoodgenomics.org/transcriptomes |
| *Castanea crenata* | Roots | Serrazina et al. 2015 | http://hardwoodgenomics.org/transcriptomes |
| *Quercus alba* | Mixed |  | http://hardwoodgenomics.org/transcriptomes |
| *Quercus rubra* | Mixed |  | http://hardwoodgenomics.org/transcriptomes |
| *Quercus robur* | All tissues | Lesur et al. 2015 | https://arachne.pierroton.inra.fr/QuercusPortal/ |
| *Alnus rubra* | Mixed |  | http://hardwoodgenomics.org/transcriptomes |
| *Alnus rhombifolia* | Mixed |  | http://hardwoodgenomics.org/transcriptomes |
| *Juglans regia* | Mixed | Martinez-Garcia et al. 2016 | http://dendrome.ucdavis.edu |
| *Juglans nigra* | Mixed |  | http://hardwoodgenomics.org/transcriptomes |
| *Corylus avellana* | All tissues | Rowley et al. 2014 | http://www.cavellanagenomeportal.com/ |
| *Fagus sylvatica* | Leaves | Lesur et al. 2015 | Genbank |
| *Fagus crenata* | Mixed | Ueno et al. 2009 | Genbank |
| *Betula platyphylla* | Mixed | Mu et al. 2012 | Genbank |
| *Nothofagus nervosa* | Leaves | Torales et al. 2012 | Genbank |
